# Direct Covalent Functionalization of H-Terminated 2D Germanane with Thiolated Molecules: Passivation and Tuning of Optoelectronic Properties

**DOI:** 10.1021/acsami.4c17152

**Published:** 2024-11-19

**Authors:** Ángel Campos-Lendinez, Jordi Faraudo, Jordi García-Antón, Xavier Sala, Jose Muñoz

**Affiliations:** † Chemistry Department, Universitat Autònoma de Barcelona, Campus UAB, Bellaterra 08193, Spain; ‡ Institut de Ciència de Materials de Barcelona (ICMAB-CSIC), Campus UAB, Bellaterra 08193, Spain

**Keywords:** germanene, 2D materials, responsive materials, molecular switches, electrodes

## Abstract

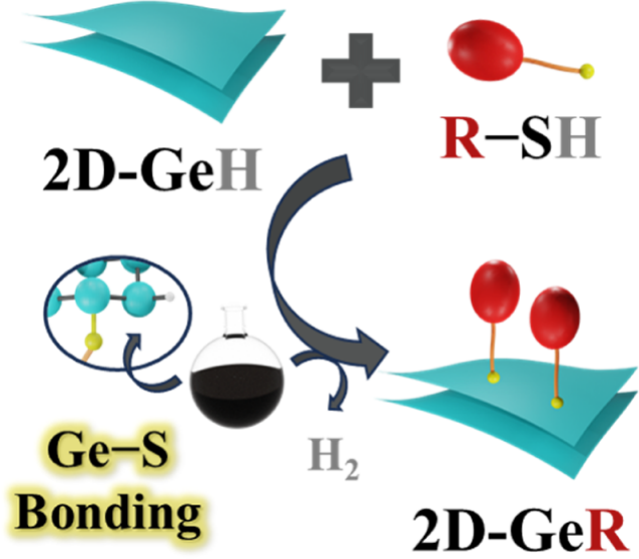

Covalent molecular functionalization allows the physicochemical properties of 2D materials to be precisely tuned and modulated on-demand. Nonetheless, research on the molecular functionalization of 2D monoelemental graphene-like materials—known as Xenes—remains scarce, being mainly restricted to a specific type of solid-state chemical reaction based on the topotactic transformation of bulkier Zintl phases. Herein, a robust and general chemical approach is reported for the direct functionalization of commercially available H-terminated 2D germanene (**2D-GeH**) with thiolated molecules (**R-SH**) via Ge–S bond formation. While the material characterization data provide direct experimental evidence of the Ge–S chemical bonding, density functional theory (DFT) calculations also predict its existence. Remarkably, the anchored thiolated molecules also favor the passivation of the 2D Xene against air oxidation, enlarging its benefits for real implementation. As a proof-of-principle, a redox-responsive molecular moiety such as 6-(ferrocenyl)hexanethiol (**Fc**_**6**_**-SH**) has been exploited to induce changes in the optoelectronic properties of the resulting **2D-GeFc**_**6**_ heterostructure by simply modulating the external bias potential, making it possible to optically and electrically read out a molecular switch on 2D Xene via implanting molecular responsiveness. Remarkably, the ON/OFF ratio has been shown to be dependent on the distance between the redox-responsive Fc moiety and the 2D Xene surface through the alkyl chain length. Overall, the reported a-la-carte molecular engineering approach provides the basis toward the rapid development of stable **2D-GeR** derivatives exhibiting molecule-programmable properties.

## 1 Introduction

The field of two-dimensional (2D) materials had an inflection point after the discovery of graphene in 2004.^1^ Its outstanding features and extensive fields of applications have ignited a growing interest in the synthesis, functionalization, and exploitation of alternative inorganic 2D materials (i2DMs), such as transition metal chalcogenides,^2−4^ transition metal carbides/nitrides (MXenes),^5,6^ 2D carbon allotropes,^7^ or monoelemental materials akin to graphene (Xenes).^8−11^ In particular, newcomers 2D Xenes have emerged as a new family of semiconducting i2DMs owing to their promising physicochemical features.^12−15^ Beyond graphene, the first generation of 2D Xenes arose with the aim to expand the family of 2D monoelemental materials belonging to the group IVA, resulting in silicene (**2D-Si**), germanene (**2D-Ge**), stanene (**2D-Sn**), and plumbene (**2D-Pb**).^16−19^ Particular attention has been devoted to the predicted properties of **2D-Ge** due to its large band gap and easily tailored optoelectronic properties.^16,20^ Nonetheless, the main challenge in the field relies on making **2D-Ge** functional for task-specific applications, being limited by its low stability and air reactivity. Further research is therefore needed to accurately develop simple functionalization methods for the synthesis of ligand-terminated forms of **2D-Ge** to prevent oxidation.

To date, ligand-terminated **2D-Ge** derivatives have been mainly realized by a specific type of topochemical transformation of Zintl-phase CaGe_2_ under harsh conditions.^21,22^ While a Zintl phase is defined as the product of a reaction between an alkaline-earth or rare-earth element with a group IV or V element (e.g., CaGe_2_),^23^ the topochemical transformation refers to a type of a solid-state reaction in which guest species are introduced into the guest solid material in a way that the morphology of precursors is retained.^24^ For example, immersing bulky CaGe_2_ into concentrated halogen acids (HX) has led to the mass production of H-terminated **2D-Ge** (**2D-GeH**), which is currently the only commercially available 2D Xene.^25^ However, the almost unexplored chemical reactivity of **2D-GeH** has hindered its direct functionalization with alternative functional R groups. Thus, the synthesis of R-terminated **2D-Ge** (**2D-GeR**) is limited to the topochemical transformation of the Zintl phase using alkyl halides (RX).^26,27^ In any case, even if this procedure is tedious, the terminal R ligand ends up being covalently bound to each Ge atom. In 2019, Hartman and co-workers described a laborious functionalization procedure for the direct modification of **2D-GeH** with RX using strong bases as alkali metal arenides.^28^ More recently, Muñoz and co-workers demonstrated the suitability of the thiol–ene click chemistry for the direct covalent functionalization of allyl-terminated **2D-Ge** (viz. **2D-GeCHCHCH_2_**) with different thiol-rich moieties via Ge–C–S bond formation.^29,30^ However, the large bond distance between the functional moiety and 2D Xene reduces the proper tunability of the material.

Among the limitless variety of ligands that can be custom-designed and synthesized with predictable functionalities, the integration of active molecular components (i.e., stimuli-responsive molecules) onto i2DMs is very appealing to tune and modulate the physicochemical properties of materials on-demand.^31−33^ However, to the best of our knowledge, the combination of active molecular components with **2D-GeH** via molecular engineering is an unexplored field that would pave the basis toward the synthesis of a new family of molecule-programmable **2D-GeR** derivatives with promising implementation in logical information processing, otherwise unattainable for their pristine counterparts.

**Scheme 1:**
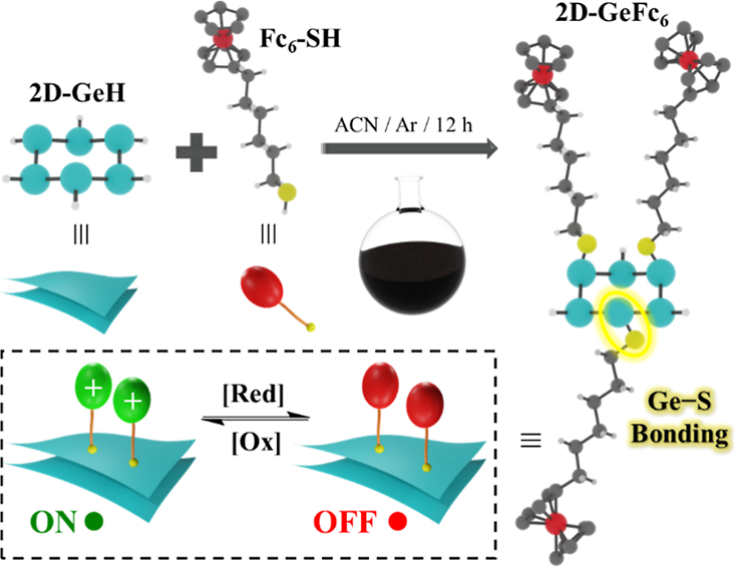
Schematic Illustration of the Direct Molecular Functionalization Approach via the Thiolation Reaction Synthesis of **2D-GeFc**_**6**_ via Ge–S bond formation by mixing **2D-GeH** with **Fc**_**6**_**-SH**. Inset: molecule-programmable features driven by modulating the oxidation/reduction ([Ox]/[Red]) bias potential.

Herein, motivated by the possibility of promoting stability and increasing the diversity of ligand functionalization of **2D-Ge** for task-specific applications, a direct covalent molecular functionalization strategy has been devised for the modification of commercially available **2D-GeH**. The reported molecular engineering approach relies on directly reacting pristine **2D-GeH** with thiolated molecules to induce the formation of a new Ge–S chemical bond (see Scheme 1 for illustration). The methodology has been inspired by taking advantage of the ability of thiol groups to passivate Ge(100) crystals^34,35^ and thus prevent oxidation. As a proof-of-principle, 6-(ferrocenyl)hexanethiol (**Fc**_**6**_**-SH**) has been utilized as a model molecular component, owing to its inherent redox-responsiveness. Interestingly, two distinguishable bistable molecular states {**2D-GeFc**_**6**_ ↔ **[2D-GeFc**_**6**_**]**^**+**^} were read (ON) and erased (OFF) by modulating the external bias potential (inputs), leading to the tuning of both the optical and electrical properties of the 2D Xene (outputs). In this last regard, the tail length of the molecular moiety has been shown to highly influence the ON/OFF ratio. Finally, the versatility of the covalent molecular approach has been extended to alternative thiolated molecules (see Figure S1 for chemical structures), while density functional theory (DFT) calculations have been run to validate the feasibility of the chemical bonding.

## 2 Experimental Section

### 2.1 Chemical and Reagents

Pristine **2D-GeH**, 6-(ferrocenyl)hexanethiol (>99%), 11-(ferrocenyl)undecanethiol (>99%), thiophenol (>99%), and high-performance liquid chromatography-grade acetonitrile (ACN) solvent were purchased from Sigma-Aldrich. Electrochemical phosphate-buffered saline (PBS) was also acquired from Sigma-Aldrich. Electrochemical aqueous solution was prepared using ultrapure water from a Milli-Q system (Millipore).

### 2.2 Instrumentation

X-ray diffraction (XRD) measurements were acquired in a Bruker AXS D8 ADVANCE diffractometer equipped with a position-sensitive detector and a curved germanium (111) primary monochromator, and the radiation used was Cu-Ka (1.5418 Å).

Fourier transform infrared (FTIR) spectra were recorded using a Bruker spectrophotometer, Alpha II model with a single reflection diamond attenuated total reflectance (ATR) module. Measurements were run in a range of 400–3000 cm^–1^.

X-ray photoelectron spectroscopy (XPS) measurements were performed with a SPECS PHOIBOS 150 hemispherical analyzer (SPECS GmbH, Berlin, Germany) at room temperature in a base pressure of 5 × 10^–10^ mbar using monochromatic Al K alpha radiation (1486.74 eV) with an excitation source operated at 300 W. The energy resolution measured by the fwhm of the Ag 3d_5/2_ peak for a sputtered silver foil was 0.62 eV.

UV–vis spectra were acquired using a V-730 JASCO spectrophotometer from 800 to 200 at a 400 nm s^–1^ scan rate (SR) using a quartz cuvette filled with 0.1 mg mL^–1^ of sample in ACN solution.

Fluorescence plots were acquired using a Cary Eclipse Fluorimeter using an excitation wavelength of 354 and 338 nm for **2D-GeH** and **2D-GeFc**_**6**_, respectively. The emission fluorescence spectra were recorded from 400 to 650 nm in a quartz fluorescence cuvette containing 0.1 mg mL^–1^ of sample in an ACN solution.

Spectro-electrochemical experiments were carried out using a quartz cuvette with a modified Teflon cap holding a Pt wire as the CE, Ag/AgCl wire as the RE, and a Pt mesh as the working electrode immersed into an ACN solution containing 0.1 M TBAPF_6_ as the electrolyte. Measurements were conducted using 0.1 mg mL^–1^ dispersions of either pristine **2D-GeH** (control) or **2D-GeFc**_**6**_ under stirring conditions (400 rpm), employing an excitation wavelength of 354 and 338 nm, respectively. Spectro-electrochemical plots were recorded from 400 to 650 nm. The time vs fluorescence intensity spectra were recorded by monitoring the emission band under oxidation or reduction conditions in pulses of 90 s.

Cyclic voltammetry (CV) and capacitance measurements were carried out with a PalmSens4 Potentiostat by using PSTrace 5.10.5604 software. The electrochemical experiments were performed using 0.1 M PBS in Milli-Q water at pH = 7.2 by immersing a platinum wire as CE, Ag/AgCl as RE, and a glassy carbon disk electrode drop casting 30 μL of sample (*C* = 1 mg mL^–1^) as WE. Capacitance plots were recorded in a range of 1 × 10^5^ to 0.1 Hz using a bias potential of 0.0 and 0.26 V for **2D-GeH** and **2D-GeFc**_**6**_ samples and 0.0 and 0.36 V for **2D-GeFc**_**11**_. The electroactive content of Fc as surface coverage (Γ) was calculated by following eqs 1 and 2(36)
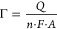
1
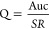
2where *Q* is the charge transferred and determined by the quotient of the integrated area under the curve (Auc) of the redox peak and the SR, *n* corresponds to the number of electrons transferred, *F* denotes the Faraday constant, and *A* signifies the electrode area immersed in the solution.

### 2.3 DFT Calculations

DFT electronic structure calculations of **2D-GePh** and **2D-GeFc**_***n***_ with different chain lengths (*n* = 2 or 6) were performed in ACN solvent. All calculations were carried out using the Gaussian 16 program, revision B.01. Two different choices of functionals and basis sets were employed, M06-L/SDD and wB97XD/cc-pVTZ, to assess the possible dependence of the results on the level of theory. The solvent was modeled with the polarizable continuum model using the integral equation formalism variant (IEFPCM), which is the default solvent model in Gaussian.

M06-L hybrid functional of Truhlar and Zhao^37^ was selected because of its broad accuracy between different elements. As basis set, the commonly used SDD basis set was selected that combines double-ζ with the Stuttgart–Dresden ECP, which reduces the cost caused by a large number of electrons, giving close agreement with experimental results.^38^ We have also considered the double hybrid wB97XD functional ,^39^ which is a dispersion-corrected highly transferable functional that could predict accurate energetics for both bonded and nonbonded interactions. As a basis set, standard polarized triple-ζ cc-pVTZ was considered.

In each case, geometry optimization and energy calculation of four structures were considered: the desired final structure (**2D-GeFc**_**2**_, **2D-GeFc**_**6**_, and **2D-GePh**), a single molecule with a thiol termination (**Fc**_**2**_**-SH**, **Fc**_**6**_**-SH**, and **Ph-SH**, respectively), the Ge structure capped with hydrogens, and a H_2_ molecule (resulting from the creation of the H atom removed from the –SH termination of each molecule and a H removed from the Ge capping). Given the energies of these partial calculations (denoted as *E*_1_, *E*_2_, *E*_3_, and *E*_4_, respectively), the Ge–S bond energy was calculated as follows (see eq 3)
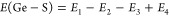
3

The model employed in the calculations describes the surface in a simplified way, considering only the Ge atoms that are first neighbors of the S atom, as done previously in the literature. In order to assess this approximation, two possible alternative models were considered for the particular case of **2D-GePh** (the system with fewer atoms), and the results were consistent with our previous calculation. The first alternative model consists in considering only a single Ge atom (the one bonded to S) capped with H atoms and the second alternative model consists in considering 13 Ge atoms that include not only first neighboring Ge but also enough Ge atoms to include three hexagonal Ge rings of the surface. The results computed using wB97XD/cc-pVTZ are shown in Table S1. These results indicate that the Ge–S bond length is not sensitive to the surface model employed. Also, we see that the *E*(Ge–S) energy (computed as described above) is sensitive to the number of Ge atoms in the model, being more negative as we add more Ge atoms to the model; therefore, our calculation probably underestimates the Ge–S bond energy.

## 3 Results and Discussion

### 3.1 Synthesis of **2D-GeFc**_**6**_ via Ge–S Bond Formation

Although this work is mainly focused on the results derived from the functionalization of pristine **2D-GeH** with **Fc**_**6**_**-SH**, the versatility of the approach has been elucidated by exploring two alternative thiolated molecular components such as 11-(ferrocenyl)undecanethiol (**Fc**_**11**_**-SH**) and thiophenol (**Ph-SH**); see Supporting Information for further details. The synthetic conditions for the covalent anchoring of **Fc**_**6**_**-SH** onto **2D-GeH** are illustrated in Scheme 1. Briefly, a 1 mg mL^–1^**2D-GeH** dispersion in deoxygenated ACN was prepared in a round flask under inert conditions. The resulting dark suspension of **2D-GeH** was sonicated for 30 min. Next, 2.5 mM **Fc**_**6**_**-SH** was added to the round flask containing the **2D-GeH** and aged for 12 h under stirring conditions in an inert (Ar) atmosphere to induce the new Ge–S chemical bond, resulting in **2D-GeFc**_**6**_.

### 3.2 Material Characterization

**Figure 1:**
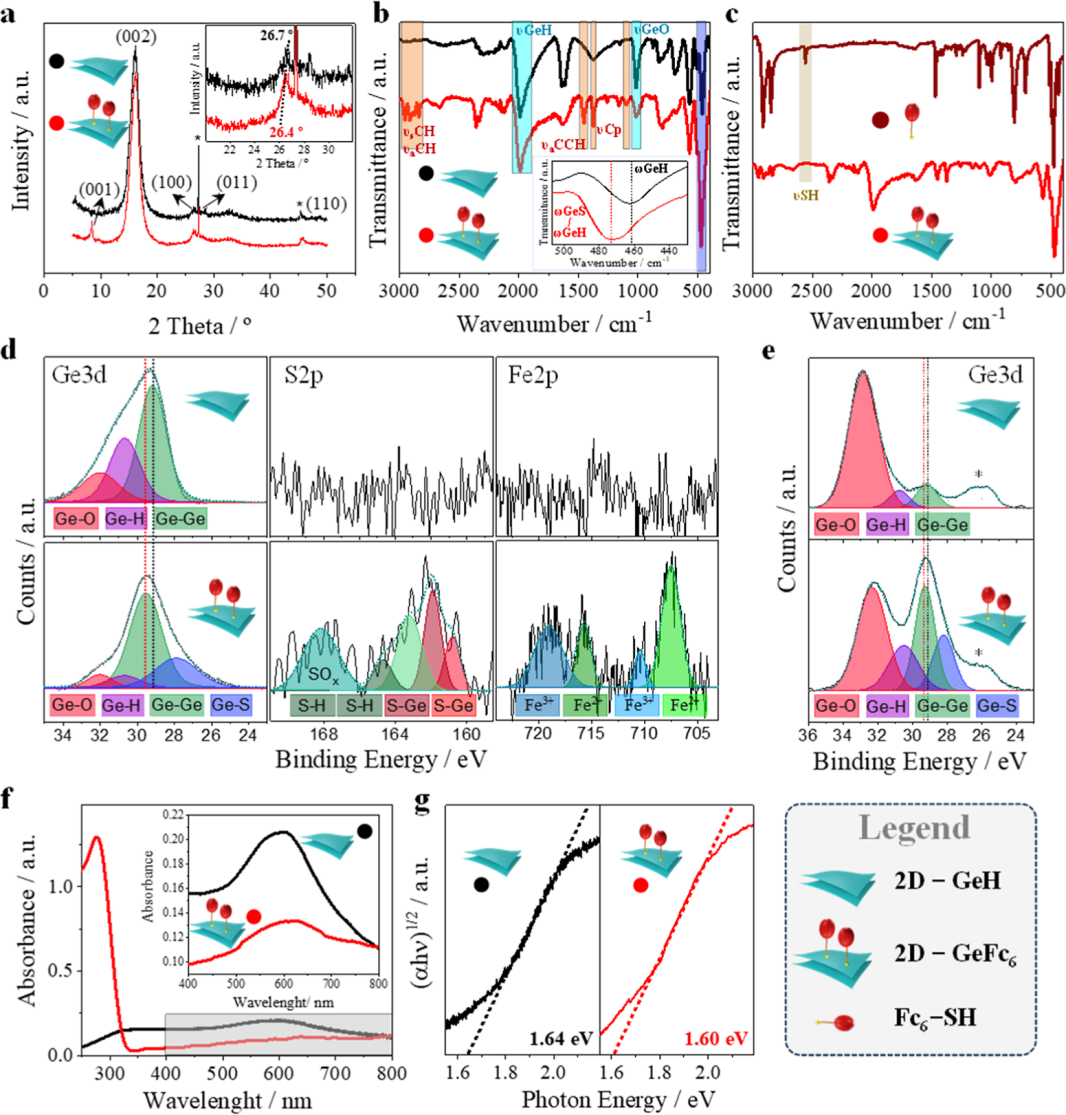
Material characterization of **2D-GeFc**_**6**_. (a) XRD and (b) FTIR spectra of pristine **2D-GeH** and **2D-GeFc**_**6**_, highlighting the main shifts observed (insets). (c) FTIR spectra of **2D-GeFc**_**6**_ and isolated **Fc**_**6**_**-SH**. (d) XPS main core-level spectra of Ge 3d, S 2p, and Fe 2p recorded for pristine **2D-GeH** (top) and **2D-GeFc**_**6**_ (bottom). (e) XPS core-level spectra of Ge 3d for pristine **2D-GeH** (top) and **2D-GeFc**_**6**_ (bottom) after 15 days under air exposure [note: XPS measurements were run on an FTO substrate, and therefore, a new contribution centered at 26.0 eV is observed (*), which corresponds to Sn 4d]. (f) UV–vis spectra of pristine **2D-GeH** and **2D-GeFc**_**6**_ with (g) the corresponding Tauc plots displaying the optical band gaps.

Both pristine **2D-GeH** and functionalized **2D-GeFc**_**6**_ were fully characterized by employing several techniques (Figure 1). First, the morphological aspect of the 2D Xene before and after functionalization was studied using transmission electron microscopy (TEM). TEM images from Figure S2 revealed a decreasing cluster size with significant delamination after functionalization with **Fc**_**6**_**-SH** molecules, suggesting that incorporation of an alkyl chain might promote a higher distance between layers. To confirm this, further studies were conducted by means of powder XRD analyses. The ligand-exchange process in Xenes is prone to develop layer disorder, which are characterized by two principal types of defects, viz., interlayer spacing (*c*-spacing) and layer shift (α-lattice).^40^Figure 1a depicts the XRD patterns of pristine **2D-GeH** and functionalized **2D-GeFc**_**6**_. Both spectra revealed the typical reflection patterns of pristine **2D-GeH** at 2θ angles of ca. 16.18°—corresponding to the (002) plane—which can be directly related to the *c*-spacing. In addition, the α-lattice parameter, which is known to vary depending on the nature of the ligand, can be calculated from the second major reflection. The reflection of the (100) plane for pristine **2D-GeH** occurs at 2θ = 26.7°. A slight shift to 2θ = 26.4° has been observed for **2D-GeFc**_**6**_, indicating that the α-lattice distance increases by 0.1 Å (from 3.3 to 3.4 Å). Contrary to pristine **2D-GeH**, an additional peak at 2θ = 8.4° was clearly observed after 2D Xene functionalization, indicating a change in the crystalline crystallization. According to the literature, this peak typically appears after the incorporation of organic ligands and corresponds to the (001) plane,^26,41^ which can be directly related to the interlayer distance (*d*-spacing). Thus, a *d*-spacing of ca. 1.05 nm can be obtained through the Bragg’s law, this value being well in line with the length of the **Fc**_**6**_**-SH** moiety. Consequently, XRD results revealed structural changes in the inherent **2D-GeH** lattice after functionalization, as well as a slight increase in the distance between sheets (*d*-spacing), suggesting the presence of molecular components covalently anchored to the 2D Xene. Then, the materials were characterized by FTIR spectroscopy by using ATR. Figure 1b shows the FTIR spectra of pristine **2D-GeH** and functionalized **2D-GeFc**_**6**_. In the recorded spectra from 3000 to 400 cm^–1^, both **2D-GeH** and **2D-GeFc**_**6**_ samples exhibited the typical Ge–H bands derived from pristine **2D-GeH** located at ca. 1990 (νGeH), 570 and 504 cm^–1^ (ωGeH), as well as the signature of Ge vacancies located between 770 and 830 cm^–1^.^42^ In addition, the band observed at ca. 987 cm^–1^ can be ascribed to the stretching modes of Ge–O (νGeO), intensity that weakened after material functionalization. Importantly, the characteristic absorption bands of **Fc**_**6**_**-SH** were clearly observed in the spectrum of **2D-GeFc**_**6**_. The fingerprint of the molecular moiety was observed at ca. 2953–2832—attributed to symmetric (ν_s_CH) and asymmetric (ν_a_CH) C–H stretching modes—1453 cm^–1^ corresponding to the in-plane C=C–H asymmetric stretching (ν_a_CCH) of the aromatic ring, and the C–H stretching vibration from cyclopentadiene (νCp) at 1376 and 1098 cm^–1^.^43−45^ Interestingly, the band ascribed to the Ge–H wagging mode (ωGeH) of pristine **2D-GeH** was significantly blue-shifted from 461 to 473 cm^–1^ after molecular functionalization (Figure 1b, inset). As previously reported in alternative ligand-terminated **2D-Ge** derivatives via Ge–C chemical bonds (e.g., **2D-GeCH**_**3**_), the resulting Ge–C wagging mode (ωGeC) contribution appears at higher wavenumbers.^22,46^ Therefore, the shift observed here must be ascribed to the Ge–S wagging mode (ωGeS), which might partially replace and mask any residual ωGeH contribution.^26^ This can be seen as a first indication of covalent Ge–S bond formation. Remarkably, the absence of the characteristic S–H stretching mode of thiol groups (νSH) reported at ca. 2550–2600 cm^–1^ points out the chemical nature of the interaction rather than a physisorption process (see Figure 1c).^47^ Accordingly, FTIR spectroscopy confirms the ferrocene termination of the germanium atoms.

The surface chemical composition of the materials was addressed by means of XPS. The XPS signal due to adventitious carbon located at 284.8 eV was used as a binding energy reference. As shown in Figure S4, the wide XPS spectra revealed a significant shift in the peak attributed to Ge 3d, while the S 2p and Fe 2p signals were detected only for **2D-GeFc**_**6**_, suggesting proper molecular functionalization. Aiming at gaining further insights into the nature of the newly formed Ge–S bond, the high-resolution XPS spectra of pristine **2D-GeH** and functionalized **2D-GeFc**_**6**_ were recorded for Ge 3d, S 2p, and Fe 2p orbitals (see Figure 1d). In line with the literature,^30^ the Ge 3d spectrum of pristine **2D-GeH** exhibited three distinct peaks at ca. 32.0, 30.7, and 29.2 eV, attributed to Ge–O, Ge–H, and Ge–Ge binding energies, respectively. After **2D-GeH** functionalization with the thiolated moiety, a new contribution was clearly observed at 27.9 eV, which is attributed to Ge–S binding energy, confirming the chemical bond formation. In addition, the shift observed in the Ge–Ge binding energy of **2D-GeFc**_**6**_ from 29.2 to 29.6 eV further corroborates the successful ligand-exchange process. Shifts in the binding energy can be directly related to charge transfer processes between metals and anchored ligands.^48,49^ Remarkably, a decrease in the Auc of the Ge–O binding energy was also revealed by the **2D-GeFc**_**6**_ sample, suggesting that its functionalization passivates the surface of the 2D Xene and therefore protects it from spontaneous oxidation, as observed in the FTIR spectra (Figure 1b). Ligand-exchange quantification was calculated by the total Auc of the Ge–S contribution, yielding 25.2% of **Fc**_**6**_**-SH**. Consequently, a Ge/S ratio of 6:2.5 was obtained, pointing out the efficient ligand-exchange substitution via the straightforward covalent molecular functionalization strategy illustrated in Scheme 1. In addition, the high-resolution XPS spectra of S 2p and Fe 2p corroborated the absence of **Fc**_**6**_**-SH** in pristine **2D-GeH**, while two pairs of peaks in both spectra were clearly identified in the **2D-GeFc**_**6**_ sample. On the one hand, the high-resolution S 2p spectrum displayed a doublet of peaks centered at 160.7 and 161.9 eV, ascribed to the Ge–S binding energy contributions of the S 2p_3/2_ and S 2p_1/2_ orbitals, respectively. This is also an indication of the covalent nature of the interaction between the **Fc**_**6**_**-SH** molecules and pristine **2D-GeH**. The second doublet was observed at 163.2 eV (S 2p_3/2_) and 164.7 eV (S 2p_1/2_), which can be attributed to nonbonded thiol groups, while the peak centered at 168.2 eV is related to the inherent oxidized S.^50^ On the other hand, the high-resolution Fe 2p spectrum also revealed the presence of Fe in the **2D-GeFc**_**6**_ sample by the two doublets centered at 707.5 and 715.6 eV and at 710.4 and 719.3 eV, corresponding to the 2p_3/2_ and 2p_1/2_ orbitals of Fe^2+^ and Fe^3+^, respectively.^51,52^ In order to demonstrate the passivation activity of thiolated molecules on **2D-GeH**, the XPS spectra of both **2D-GeH** and **2D-GeFc** were acquired after 15 days under air exposure. As shown in Figure 1e, after 15 days, the inherent XPS fingerprint of **2D-GeH** almost disappeared, leading to a %O as high as 77%. Contrary, the %O for **2D-GeFc**_**6**_ was found to be 40%, demonstrating that the devised molecular approach is a powerful strategy to enlarge the lifetime of the 2D Xene.

Finally, the optical properties of both pristine **2D-GeH** and functionalized **2D-GeFc**_**6**_ were characterized by UV–vis spectroscopy. Figure 1f shows the UV–vis spectra in a range of 800 to 250 nm using a 1 mg mL^–1^ aqueous suspension of sample. The UV–vis spectra of pristine **2D-GeH** displayed a maximum absorption band at 602 nm, attributed to the π–π* transitions.^16,53^ Importantly, **2D-GeFc**_**6**_ presented a red shift in this band to 663 nm owing to the ligand-exchange reaction. This led to a notable band gap shift in the 2D Xene from 1.64 to 1.60 eV, as demonstrated by the TAUC plots presented in Figure 1g. This result supports once again the proper covalent Ge–S bond formation since it is well-known that the band gap of **2D-Ge** can be tuned by tailoring the nature of the terminal ligand covalently anchored to the **2D-GeH**.^22,29^ Moreover, an additional band with a maximum centered at 277 nm was clearly observed in the UV–vis spectra of **2D-GeH**, which corresponds to the π–π* transitions of the Fc groups.^54^

### 3.3 Suitability of the Chemical Bonding via DFT Calculations

**Figure 2:**
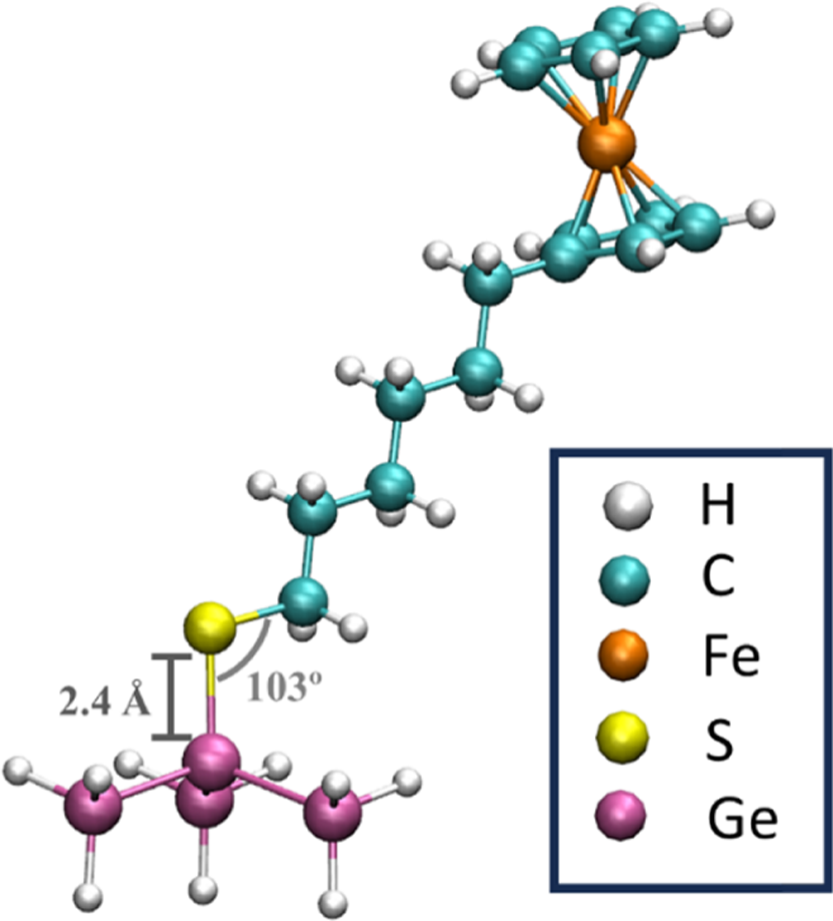
Theoretical modeling of the **2D-GeFc**_**6**_ structure. DFT-optimized structure (M06-L/SDD) in CPK representation for **2D-GeFc**_**6**_. The color code is indicated in the figure. Image made with VMD.^60^

The Ge–S bond was studied by performing DFT electronic structure calculations of **2D-GeFc**_**6**_ in ACN via Gaussian 16^55^ with two different choices of functionals and basis sets (M06-L/SDD and wB97XD/cc-pVTZ).^56,57^ The ACN solvent was modeled implicitly using the IEFPCM formalism.^58^ The modeling of the Ge–S bond was done (as in previous DFT calculations of similar systems^59^) including Ge atoms that are first neighbors of the Ge bonded to S (see Figure 2), which are subsequently capped with H atoms. Further, isolated molecules and other fragments were also simulated in order to evaluate the formation energy of the Ge–S bond (see Supporting Information for more details). As a result, a stable Ge–S bond was observed independently of the level of DFT theory employed with bond energies of −8.5 and −5.6 kcal mol^–1^ for M06-L/SDD and wB97XD/cc-pVTZ, respectively. The Ge–S bond length was found to be ∼2.4 Å with a Ge–S–C angle of 103°, as shown in Figure 2.

### 3.4 Molecule- Programmable (Opto)electronic Properties

Having verified theoretically and experimentally the successful direct molecular functionalization approach via Ge–S bond formation, the next step was focused on exploiting the applicability of the resulting **2D-GeFc**_**6**_ with implanted molecular properties for programming its optoelectronic properties on-demand. In particular, the optoelectronic properties of **2D-GeFc**_**6**_ were modulated by taking advantage of the inherent redox-responsive features of the anchored molecular moiety. Thus, the implanted molecular redox responsiveness (inputs) has been exploited to monitor a bistable molecular switch with either optical or electrical readouts (outputs), leading to molecule-programmable 2D Xene.

#### 3.4.1 Modulation of the Optical Properties of **2D-GeFc**_**6**_ (Electrical Input–Optical Output)

**Figure 3:**
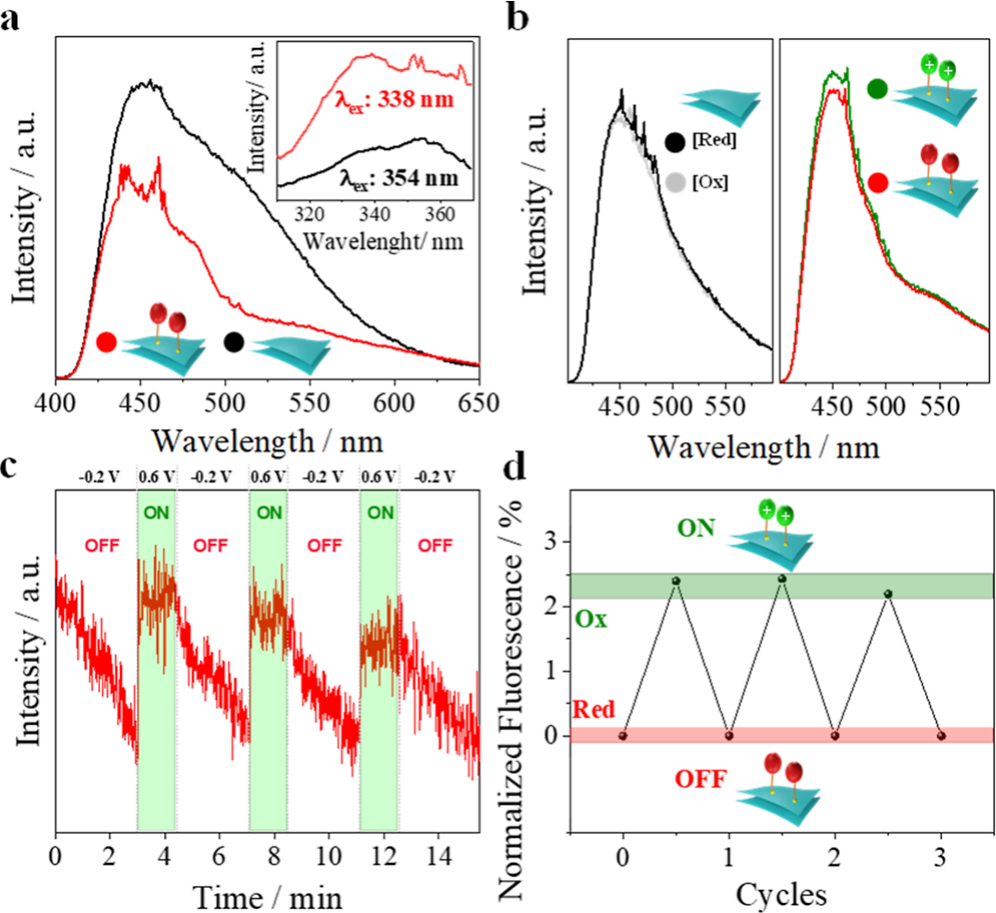
Molecule-programmable **2D-GeFc**_**6**_: electrical input–optical output. (a) Excitation (inset) and emission fluorescence spectra recorded for pristine **2D-GeH** and **2D-GeFc**_**6**_. (b) Spectro-electrochemical emission spectra of **2D-GeH** (left) and **2D-GeFc**_**6**_ (right) using oxidation (+0.6 V) and reduction (−0.2 V) bias potentials. (c) Time vs fluorescence intensity experiment for **2D-GeFc**_**6**_ by monitoring the emission band at 452 nm through modulating the bias potential from +0.6 V (**2D-GeFc**_**6**_ → **[2D-GeFc**_**6**_**]**^**+**^) to −0.2 V (**[2D-GeFc**_**6**_**]**^**+**^ → **2D-GeFc**_**6**_) in pulses of 90 s, with (d) the resulting normalized fluorescence spectra with the ON/OFF cycles. Spectro-electrochemical measurements were run in a three-electrode configuration cell made of quartz filled with 0.1 M PBS (pH 7.2) as the electrolyte.

First, the implanted molecular responsiveness of the redox-responsive ligand was utilized to tune the intrinsic fluorescence features of 2D Xene. Fluorescence spectroscopy measurements are shown in Figure 3a. The emission spectra of both pristine **2D-GeH** and functionalized **2D-GeFc**_**6**_ were centered at λ_em_ = 452 nm, while the excitation wavelength was shifted from λ_ex_ = 354 nm to λ_ex_ = 338 nm after molecular functionalization (Figure 3a, inset), in line with the band gap change observed in Figure 1g. The highest the electronic band gap, the lowest the λ_ex_. In addition, a quenching in the fluorescence intensity as high as 35% was observed for functionalized **2D-GeFc**_**6**_ with respect to the pristine **2D-GeH** sample. This quenching mechanism must be promoted by a nonradiative recombination from the conduction band owing to the fact that thiol groups can act as hole trap states when are covalently attached, resulting in a reduction of the fluorescence intensity.^61^ This result points out the efficiency of the ligand-exchange process and the relevant tunability of the fluorescence properties of **2D-GeH** once it is covalently functionalized via Ge–S bond formation.

Subsequently, a spectro-electrochemical experiment was performed to study whether the fluorescence features of the **2D-GeFc**_**6**_ can also be modulated by manipulating the oxidation state of the iron core of the molecular moiety through a redox-driven bistable molecular switch on the 2D Xene (**2D-GeFc**_**6**_ ↔ **[2D-GeFc**_**6**_**]**^**+**^). As shown in Figure 3b, the fluorescence intensity of **2D-GeFc**_**6**_ slightly changes up to 3% depending on the redox potential applied to either oxidize (+0.6 V) or reduce (−0.2 V) the Fc moiety. Herein, an increase in the fluorescence intensity was observed after oxidizing the iron core of the ligand, inducing the reaction: **2D-GeFc**_**6**_ → **[2D-GeFc**_**6**_**]**^**+**^. Bearing in mind that two possible events can derive from the fluorescence quenching mechanism as a dependence of the redox state of electroactive groups—electron donor or acceptor,^62^ such an intensity increase suggests that the photoinduced electron transfer is more favored when Fc acts as a donor group (**2D-GeFc**_**6**_, state OFF) rather than as an acceptor group (**[2D-GeFc**_**6**_**]**^**+**^, state ON).^63^ Importantly, no change in the fluorescence intensity of pristine **2D-GeH** (control experiment) was observed after manipulating the redox potential because of the lack of electroactive groups (Figure S3). To further verify this small change in the fluorescence properties of **2D-GeFc**_**6**_, the reversibility of the redox-driven bistable molecular switch was also interrogated. Figure 3c depicts the time vs fluorescence intensity plot of **2D-GeFc**_**6**_, in which a reversible and stable fluorescent switch with two distinguishable optical states can be clearly observed, demonstrating that the small changes observed in the fluorescence intensity with regard to the applied bias potential are consistent over time. In addition, while a quick switch in the fluorescence intensity (τ_ON_ = 0.18 s) was obtained during the oxidation process (bias potential: +0.6 V), the quenching process during the reduction process (bias potential: −0.2 V) was significantly slower (τ_OFF_ = 158 s). According to this data, the hole injection mechanism seems to be favored during the oxidation process from **2D-GeFc**_**6**_ to **[2D-GeFc**_**6**_**]**^**+**^, while the electron injection mechanism is hindered during the reduction process from **[2D-GeFc**_**6**_**]**^**+**^ to **2D-GeFc**_**6**_.^64^ As a result, a stable and reversible bistable molecular switch with two distinguishable ON/OFF states was electrically driven and optically read out using a molecule-programmable 2D Xene synthesized via a direct Ge–S bond formation (Figure 3d).

#### 3.4.2 Modulation of the Electrochemical Properties of **2D-GeFc**_**6**_ (Electrical Input–Electrical Output)

**Figure 4:**
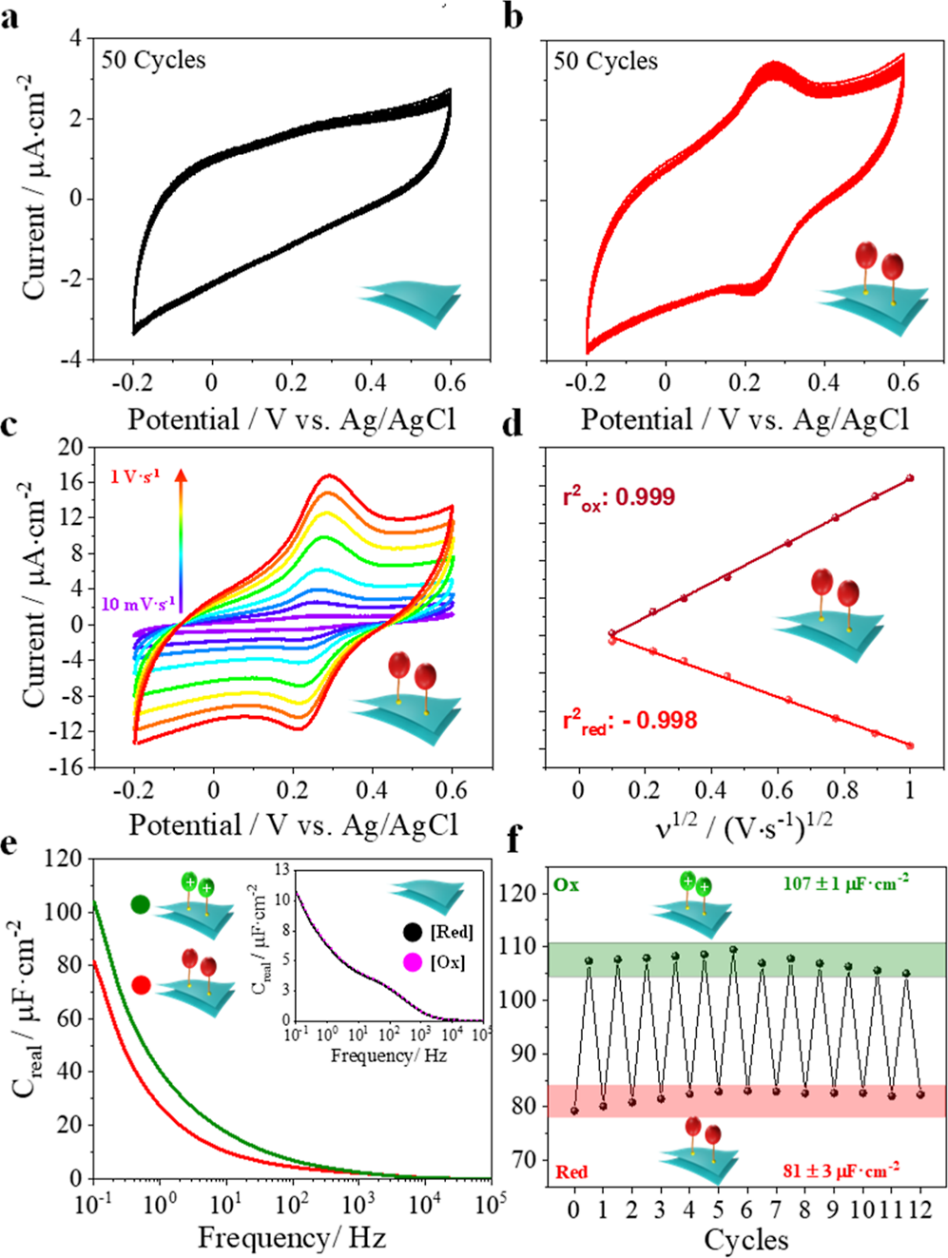
Molecule-programmable **2D-GeFc**_**6**_: electrical input–electrical output. Cyclic voltammograms over 50 cycles for (a) pristine **2D-GeH** and (b) **2D-GeFc**_**6**_. (c) **2D-GeFc**_**6**_ CVs at different SRs (0.01, 0.05, 0.1, 0.25, 0.5, 0.75, and 1 V s^–1^), and (d) current vs SR plots. (e) Bode plot displaying capacitance vs logarithm of frequency at different oxidation (+0.26 V) and reduction (−0.2 V) bias potentials for pristine **2D-GeH** (inset) and **2D-GeFc**_**6**_, with (f) its respectively 12 successive capacitance redox cycles. Electrochemical experiments were run in a three-electrode configuration cell filled with 0.1 M PBS at pH 7.2.

Beyond their optical properties, the electrochemical properties of **2D-GeFc**_**6**_ were also explored by taking full advantage of the redox responsiveness of the ligand-terminated group. To this end, a fixed amount of **2D-GeFc**_**6**_ was drop-cast onto a conventional glassy carbon electrode and exposed to a PBS solution at pH 7.2. Figure 4a,b displays the voltammetric behavior of pristine **2D-GeH** and functionalized **2D-GeFc**_**6**_ over 50 consecutive cycles. As expected, the electrochemical performance of pristine **2D-GeH** just resulted in non-Faradaic currents (Figure 4a), while **2D-GeFc**_**6**_ notably displayed a pair of well-defined anodic and cathodic peaks centered at 269 and 224 mV (vs Ag/AgCl), respectively, which might correspond to a Fc/Fc^+^ redox couple from the iron core (Figure 4b). Importantly, a peak-to-peak separation value (Δ*E*) as low as 54 mV was yielded, which must be attributed to the high confinement of the molecular moieties on the semiconductor surface. The surface coverage (Γ) was calculated by means of CV^65^ and estimated to be 3.89 × 10^–9^ mol cm^2^. This value demonstrates a notable high surface decoration via a direct covalent molecular functionalization approach. Remarkably, a lack of fatigue after 50 consecutive cycles was observed, as demonstrated by the almost unaltered current intensity signal, which evidence the outstanding robustness of the system as expected by a covalently anchored ligand. In addition, different SR vs current intensity measurements were also conducted for **2D-GeFc**_**6**_ in order to corroborate the proper confinement of the molecular moieties (Figure 4c). As shown in Figure 4d, an outstanding linear relationship with the square root of the SRs for both anodic (*r*^2^ = 0.999) and cathodic (*r*^2^ = 0.998) peaks was achieved, indicating a reversible diffusion-controlled process for the Fc/Fc^+^ redox pair.^66,67^

Afterward, the suitability of electrochemically monitoring two different electrical states was elucidated by means of electrochemical impedance spectroscopy (EIS), in which the resulting electrical output signals were acquired through the real part of the interfacial complex capacitance (*C*_re_) in the frequency range of 0.1 Hz to 100 kHz. This parameter is known to be directly related to the density of charge accumulated at the electrode/electrolyte interface.^68^ As shown in the Bode plot of Figure 4e, two well-distinguished electrical states were reached before (bias potential: 0.0 V) and after (bias potential: +0.3 V) the redox peaks, leading to a state-to-state gap (Δ*C*_re_) of 22.0 μF cm^–2^ in the low-frequency regime. Importantly, the control experiment carried out using pristine **2D-GeH** revealed unaltered *C*_re_ changes at the electrode/electrolyte interface, making it possible to ascribe the aforementioned capacitive changes of **2D-GeFc**_**6**_ to the different charge states led by the ligand-terminated group. Finally, the reversibility of the system was also interrogated by applying successive oxidation/reduction pulses over 12 consecutive cycles (Figure 4f), demonstrating the excellent stability and robustness of two electrical molecular states: **[2D-GeFc**_**6**_**]**^**+**^ (state ON) and **2D-GeFc**_**6**_ (state OFF). All in all, such capacitive changes, together with the reversibility and stability of the system upon manipulating the DC bias voltage (ON/OFF), demonstrate the feasibility of the EIS technique to electrically monitor a bistable molecular switch through molecular programming of a 2D Xene with an electroactive ligand-terminal group as Fc. Importantly, this approach is especially appealing, owing to the electrical nature of both the input and the output signals, which can be easily integrated with current technologies.

### 3.5 Versatility of the Ge–S Bond Formation

The versatility of the chemical approach was elucidated by utilizing two alternative thiolated molecules, **Fc**_**11**_**-SH** and **Ph-SH**, under the same synthetic conditions, resulting in **2D-GeFc**_**11**_ and **2D-GePh**, respectively. As shown in Figure S5, the FTIR spectrum exhibited the same blue-shift in the band located between 460 and 470 cm^–1^ observed in **2D-GeFc**_**6**_, which must be attributed to the ωGeS contribution. In addition, the strong ν_GeO_ stretching band of pristine **2D-GeH** was notably weakened after anchoring the thiolated molecules, suggesting passivation of the surface. Further, the ν_SH_ stretching band displayed by the raw thiolated molecules centered at 2550 cm^–1^ totally disappeared after **2D-GeH** functionalization (Figure S6), discarding any hypothetical physisorption process. In addition, Figure S7 presents the Ge 3d core-level spectra of both **2D-GeFc**_**11**_ and **2D-GePh**, which also exhibited the new Ge–S contribution at ∼28 eV, together with a shift in the Ge–Ge contribution toward higher binding energies (see Table S2 for further details). This reinforces the assignment made for the Ge–S peak in the **2D-GeFc**_**6**_ material (see Figure 1d), corroborating once again the chemical nature of the bonding. As shown in Figure S8, the optical features of pristine **2D-GeH** were tuned after molecular functionalization, and the determined band gaps were observed to be a red shift from 1.64 to 1.60, 1.58, and 1.61 eV for **2D-GeFc**_**6**_, **2D-GeFc**_**11**_, and **2D-GePh**, respectively. Those changes in the band gap can be considered to be a clear signal of successful covalent functionalization. Consequently, the material characterization data of **2D-GeFc**_**11**_ and **2D-GePh** align well with the results obtained by **2D-GeFc**_**6**_. Importantly, **2D-GePh** DFT calculations were also performed (Figure S9), confirming the Ge–S bonding again. Comparing the results obtained for **2D-GeFc**_**6**_, the predicted Ge–S bond distance remained unaltered, while the bond energy was slightly more favorable for **2D-GePh** (−11.5 and −6.7 kcal mol^–1^ for M06-L/SDD and wB97XD/cc-pVTZ levels of theory, respectively).

Lastly, considering the redox-responsive properties of the **GeFc**_**11**_**-SH** moiety, the electrochemical performance of the resulting **2D-GeFc**_**11**_ was also evaluated. The cyclic voltammograms in Figure S10 clearly verified the presence of the redox-responsive moiety with oxidation (Fe^2+^/Fe^3+^) and reduction (Fe^3+^/Fe^2+^) peaks located at 382 and 99 mV, respectively. From CV, an estimated Γ of 1.01 × 10^–9^ mol cm^–2^ was obtained for **2D-GeFc**_**11**_, in line with the one yielded by **2D-GeFc**_**6**_ (Γ = 3.89 × 10^–9^ mol cm^–2^). Finally, EIS measurements were run in order to read out two electrochemical states by means of *C*_dl_, resulting in a Δ*C*_dl_ value of 12.5 μF cm^–2^ (Figure S11a). In addition, the **2D-GeFc**_**11**_ sample was subjected to several redox cycles by modulating the bias potential, demonstrating excellent reversibility (Figure S11a). The outstanding molecular switchability can be ascribed to the robustness of covalent anchoring. Comparing to the findings obtained by **2D-GeFc**_**6**_—which contains an alkyl chain of 6 carbons, rather than 11—the **2D-GeFc**_**11**_ displayed a lower Δ*C*_dl_ value (12.5 vs 22.0 μF cm^–2^, see Figure S12), which can be ascribed to the larger distance of the redox center to the electrode surface. As previously reported, the length of the alkyl chain drastically influences the electrochemical behavior of Fc from a molecular switch point of view.^52,69^

## 4 Conclusions

In summary, a general molecular engineering approach has been devised for the direct covalent functionalization of commercially available **2D-GeH** with different thiolated molecular components (**R-SH**) via Ge–S bond formation, resulting in a new library of **high-stable 2D-GeR** derivatives. As a proof-of-principle, a thiol-rich active molecular component such as 6-(ferrocenyl) hexanethiol (**Fc**_**6**_**-SH**) has been utilized by taking advantage of its inherent redox-responsive features. After an accurate material characterization and DFT calculations, the nature of the chemical bonding has been demonstrated, also elucidating the passivation effect of thiolated molecules on **2D-Ge** to enlarge material stability under ambient conditions. As a result, a **2D-GeFc**_**6**_ heterostructure with implanted molecular responsiveness has been successfully synthesized via direct covalent bonding. Both the optical and electrical properties of **2D-GeFc**_**6**_ (outputs) have been tuned on-demand by simply modulating the external bias potential (input). This allows for the possibility to write (ON) and erase (OFF) a redox-driven molecular switch. In addition, the ON/OFF ratio has been shown to be dependent on the length of the Fc alkyl tail.

The versatility of the approach has been demonstrated by covalently grafting two additional thiolated molecules, such as 11-(ferrocenyl)undecanethiol (**Fc**_**11**_**-SH**) and thiophenol (**Ph-SH**) under the same synthetic conditions. The resulting **2D-GeFc**_**11**_ and **2D-GePh** heterostructures have been fully characterized, and the findings further support the new Ge–S chemical bond. Thus, the reported molecular engineering approach provides a general strategy for broadening the library of **2D-GeR** derivatives to be implemented in yet unexplored fields (i.e., logical information processing), otherwise inaccessible for the pristine **2D-GeH** counterpart. Finally, this work is supposed to be a nucleus for the custom preparation of molecule-programmable 2D Xenes by simply tailoring the responsiveness of the thiolated active ligand for (opto)electronics approaches while enlarging material stability.

## Supporting Information Available

Illustration of the thiolated molecules explored; TEM images; fluorescence measurements (control); XPS spectra; FTIR spectra; optical band gap; additional electrochemical data; and additional DFT calculations (PDF)
