# Supplementary material for: Direct Covalent Functionalization of H-Terminated 2D Germanane with Thiolated Molecules: Passivation and Tuning of Optoelectronic Properties
Source: ACS Appl Mater Interfaces. 2024 Nov 19;16(48):66280–9. doi: 10.1021/acsami.4c17152 (PMC11622187; doi:10.1021/acsami.4c17152)
Supplement: Supplementary file 1 — am4c17152_si_001.pdf [file am4c17152_si_001.pdf]

Supporting Information

Direct Covalent Functionalization of H-Terminated 2D Germanane with Thiolated Molecules: Passivation and Tuning Optoelectronic Properties

Ángel Campos-Lendínez,[a] Jordi Faraudo,[b] Jordi García-Antón,[a] Xavier Sala,[a] Jose Muñoz[a,*]

[a]Chemistry Department, Universitat Autònoma de Barcelona, Campus UAB, 08193 Bellaterra, Spain

[b]Institut de Ciència de Materials de Barcelona (ICMAB-CSIC), Campus UAB, 08193 Bellaterra, Spain

* E-mail: JoseMaria.Munoz@uab.cat

1. Supporting Figures

Figure S1: Illustration of the three thiolated molecules explored........................... S2
Figure S2: TEM images of pristine **2D–GeH** and **2D–GeFc$_6$**........................... S2
Figure S3: Time *vs.* fluorescence intensity experiment for pristine **2D–GeH** (control)... S3
Figure S4: Wide XPS spectra of pristine **2D–GeH** and **2D–GeFc$_6$**...................... S3
Figure S5: FTIR spectra of pristine **2D–GeH**, **2D–GeFc$_{11}$** and **2D–GePh** S4
Figure S6: FTIR spectra comparing heterostructures with molecular precursors.......... S4
Figure S7: High resolution core level spectra of Ge 3d of **2D–GeFc$_{11}$** and **2D–GePh**... S5
Figure S8: Optical properties of **2D–GeFc$_{11}$** and **2D–GePh**...................................... S6
Figure S9: DFT optimized structures of **2D–GeFc$_2$**, **2D–GeFc$_6$** and **2D-GePh**............ S6
Figure S10: CV of pristine **2D–GeH** and **2D–GeFc$_{11}$**.. S7
Figure S11: EIS performance of **2D–GeFc$_{11}$** at different bias potentials........................ S7
Figure S12: Cyclability comparison of **2D–GeFc$_6$** and **2D–GeFc$_{11}$**................................ S8

2. Supporting Tables

Table S2: DFT results for **2D–GePh** considering different models for the surface....... S8
Table S1: Ge 3d binding energies for the different **2D–GeR** heterostructures............. S8

1. Supporting Figures

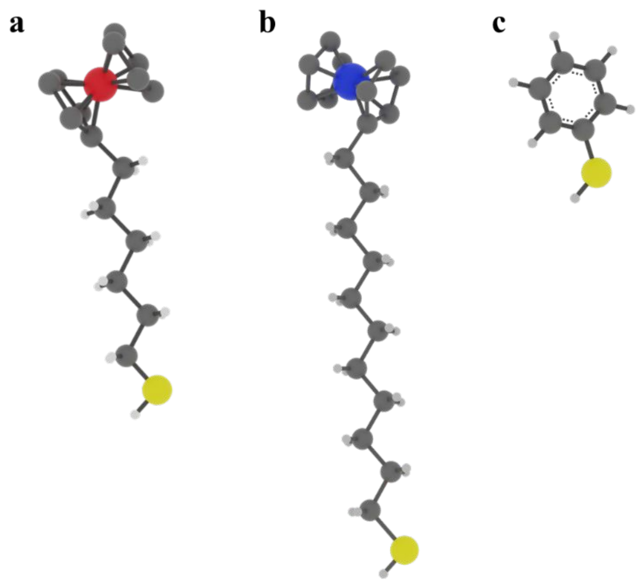

Figure S1. Illustration of the three thiolated molecules (**a)** 6-(ferrocenyl)hexanethiol, **Fc_6-SH**, **b)** 11-(ferrocenyl)unadecanethiol, **Fc_{11}-SH** and **c)** thiophenol, **Ph-SH**) employed for functionalizing **2D–GeH** *via* Ge–S bond formation, resulting in **2D–GeFc_6**, **2D–GeFc_{11}**, and **2D–GePh** heterostructures, respectively Remarkably, the thiolated molecules contain an alkyl chain with different carbon numbers (n = 0, 6 or 11).

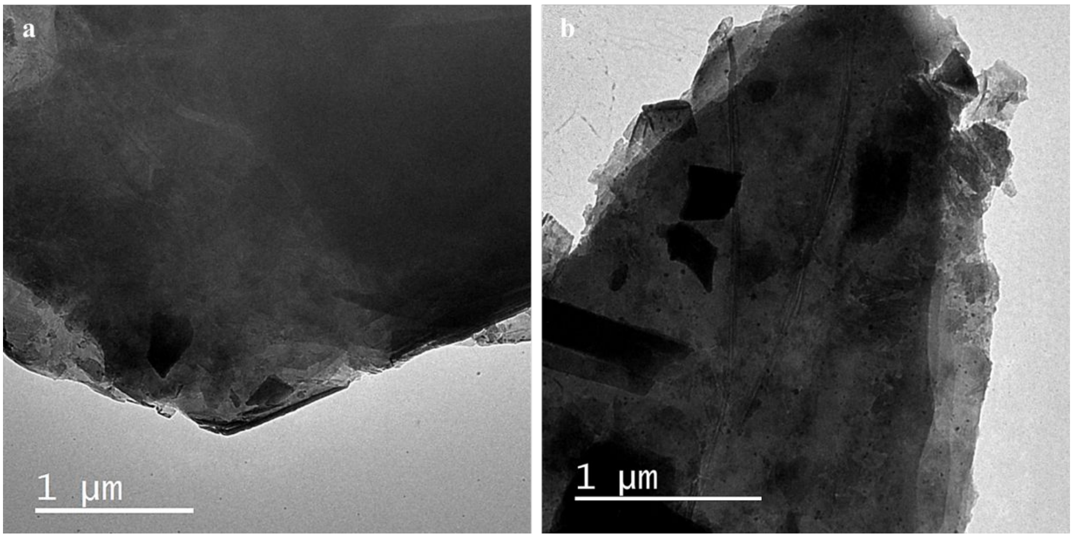

Figure S2. TEM images of **a)** pristine **2D–GeH** and **b) 2D–GeFc_6**.

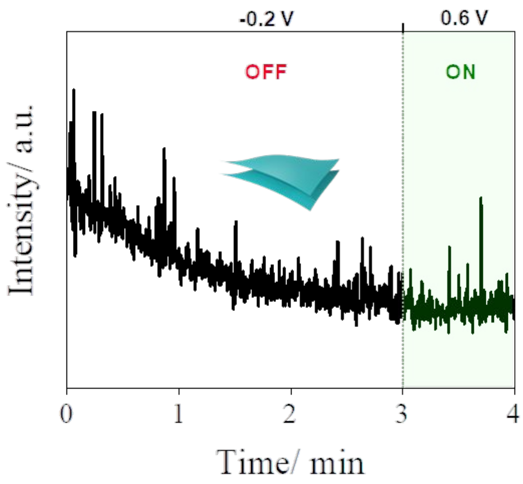

Figure S3. Time *vs.* fluorescence intensity experiment for pristine **2D–GeH** by monitoring the emission band at 452 nm using different bias potentials (+0.6 V and –0.2 V). This spectro-electrochemical control experiment demonstrates that an electroactive group is needed to trigger the optical properties of the material.

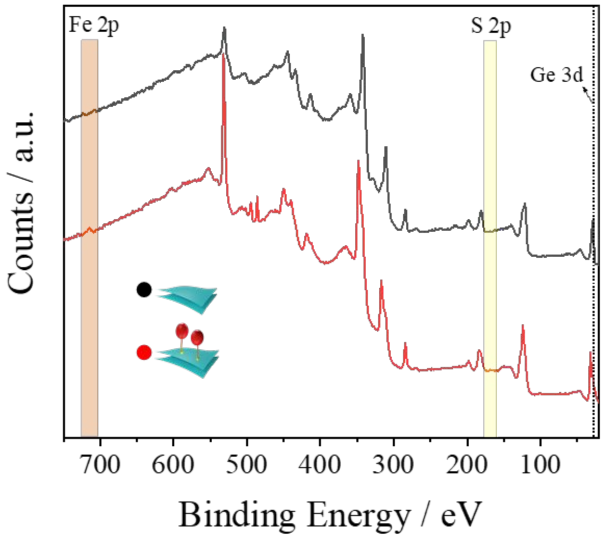

Figure S4. Wide XPS spectra of pristine **2D–GeH** and functionalized **2D–GeFc$_6$**, highlighting the shift observed for the Ge 3d contribution, as well as the contributions of Fe 2p and S 2p observed after molecular functionalization.

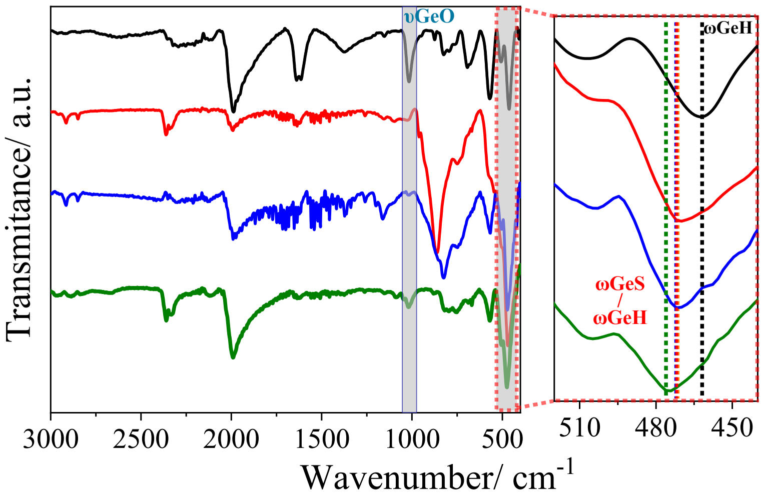

Figure S5. FTIR spectra of pristine **2D–GeH** (black), **2D–GeFc$_{11}$** (blue) and **2D–GePh** (green). The two functionalized 2D–Ge derivatives presented bands around 2900 cm^{-1}, corresponding to the stretching vibrations of C–C and C=C, suggesting the presence of organic molecules. In addition, the strong Ge–O stretching band of pristine **2D–GeH** was notably weakened after the anchoring of thiolated molecules, while its inherent Ge–H band was red-shifted owing to the formation of a new chemical bond (Ge–S).

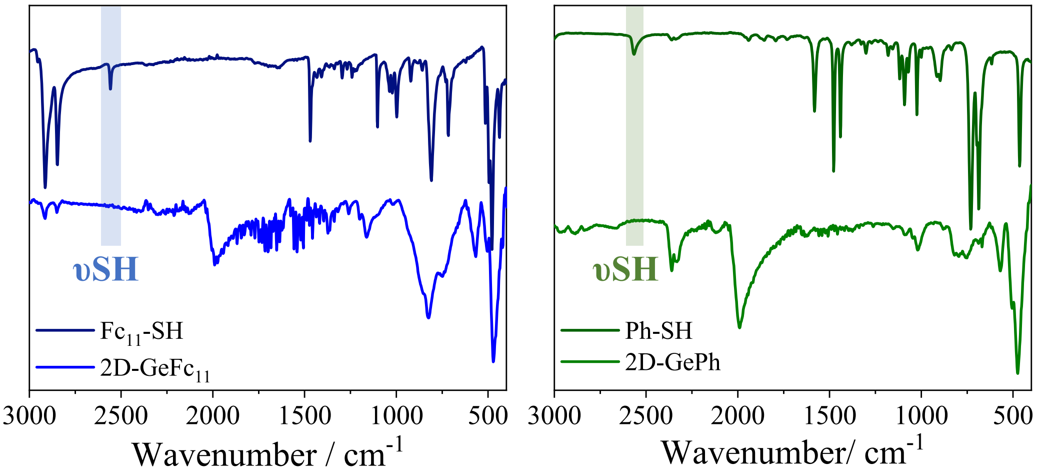

Figure S6. FTIR spectra comparing the thiolated precursors (**Fc$_{11}$–SH** and **Ph–SH**) with the resulting functionalized **2D–GeFc$_{11}$** (left) and **2D–GePh** (right). Importantly, the vibration band at ca. 2550 cm^{-1} totally disappeared after material functionalization, demonstrating that the thiolated molecules were chemically bonded to the pristine **2D–GeH** rather than physisorbed.

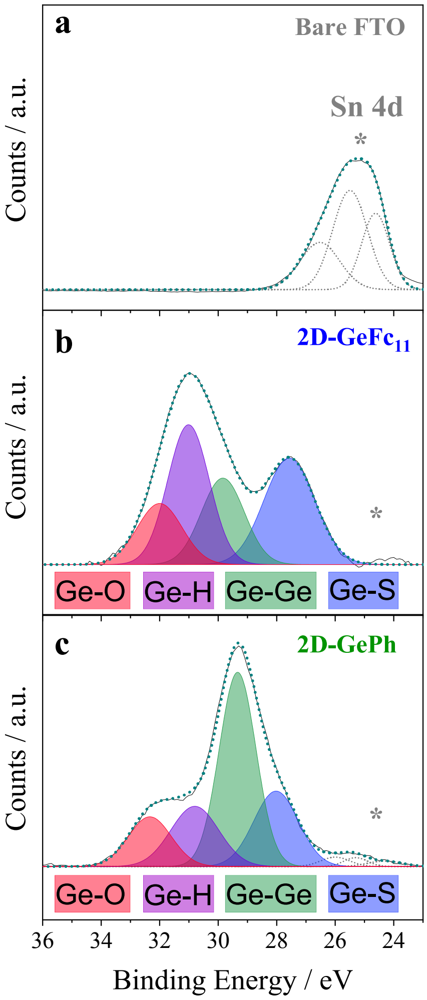

Figure S7. High-resolution core-level spectra of Ge 3d for **a)** raw FTO substrate (control), **b) 2D–GeFc$_{11}$**, and **c) 2D–GePh**. In all cases, an additional contribution centred around □ 28 eV was found, which is associated with the new Ge–S chemical bond. Note: Since measurements were done by drop-casting the materials on an FTO surface, its inherent Sn 4d contribution (*) centred at 26.0 eV can be observed.

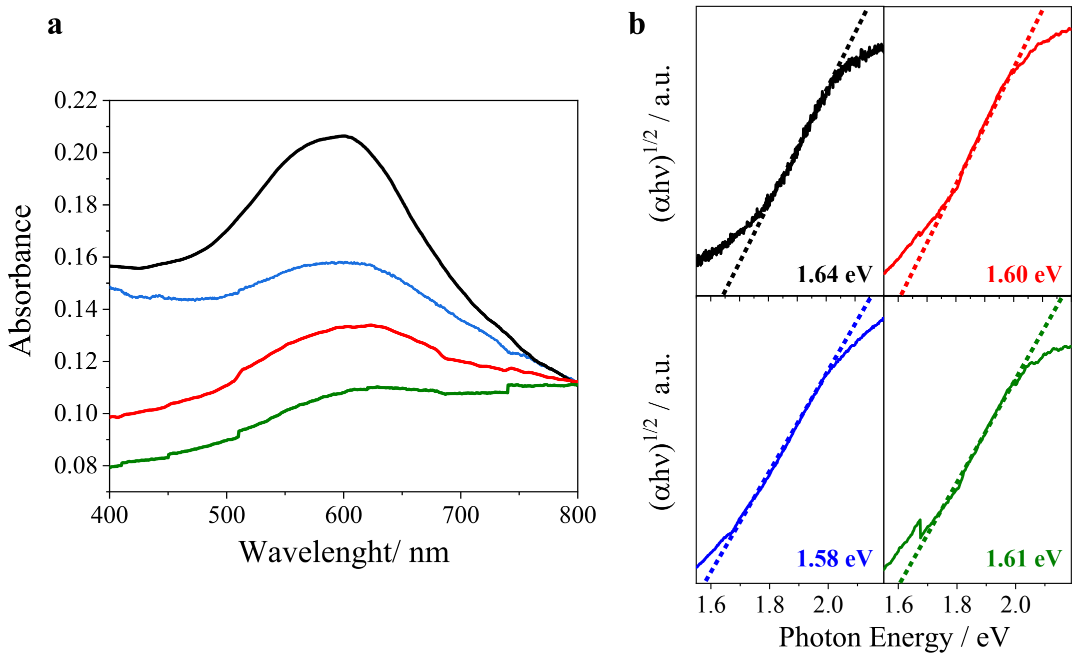

Figure S8. Optical properties of pristine **2D–GeH** (black), **2D–GeFc$_{11}$** (blue) and **2D–GePh** (green) showing the **a)** UV-vis spectra with **b)** their corresponding TAUC plots, highlighting the resulting optical band gaps. Importantly, the pristine **2D–GeH** band gap changed after material functionalization, which is a clear indicator of a covalent modification.

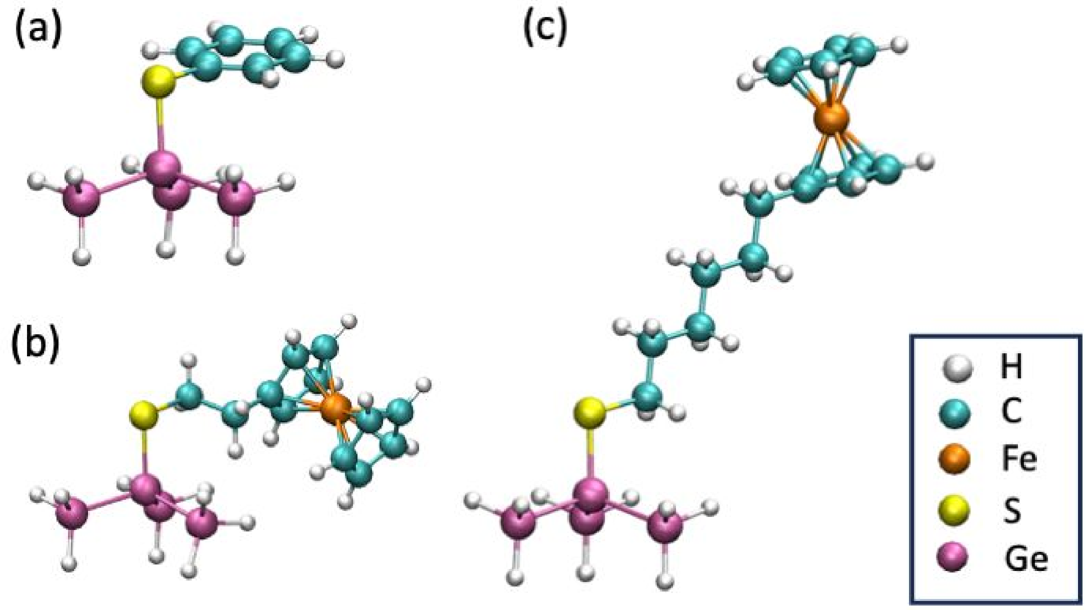

Figure S9. DFT optimized structures considered in this work shown in CPK representation: a) **2D-GePh**, b) **2D–GeFc$_2$** and c) **2D–GeFc$_6$**. The color code is indicated in the figure. Image made with VMD.

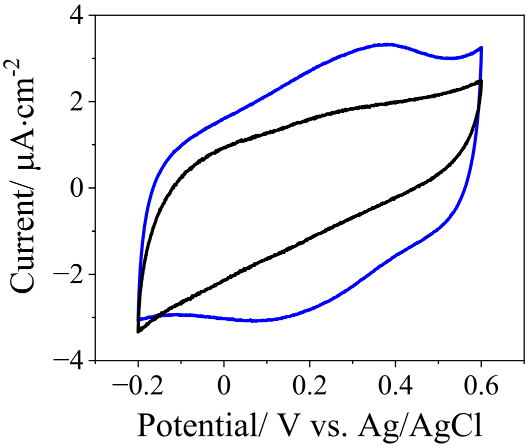

Figure S10. Cyclic voltammograms of pristine **2D–GeH** (black line) and **2D–GeFc$_{11}$** (blue line), displaying the inherent Fe^{2+}/Fe^{3+} redox process.

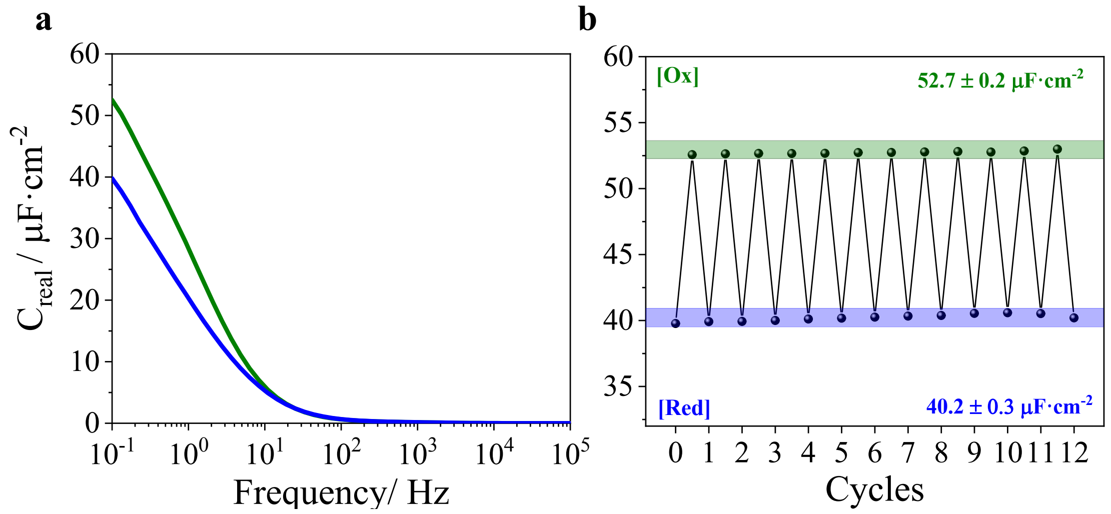

Figure S11. EIS performance of **2D–GeFc$_{11}$**: **a)** Capacitance *vs*. frequency spectra at different oxidation (+ 0.36 V) and reduction (– 0.2 V) bias potentials, and **b)** stability test after 12 successive redox cycles.

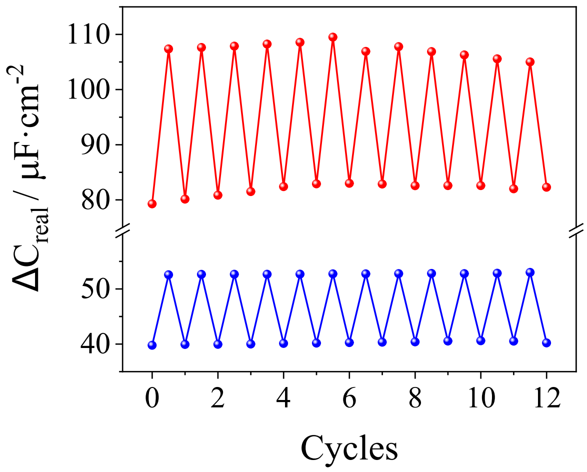

Figure S12. Comparison of the molecular switching capabilities of **2D–GeFc$_6$** (red) and **2D–GeFc$_{11}$** (blue) after 12 successive redox cycles, demonstrating the key effect of the distance of the redox-active moiety (Fc) to the electrode surface through the alkyl chain length.

2. Supporting Tables

Table S1. Comparison of DFT results for **2D–GePh** considering different models for the surface.

**Surface Model**	**DFT values**		
	**E(Ge–S)**	**Ge–S bond length**	**Ge–S–C angle**
Surface with 1 Ge	-3.4 kcal/mol	2.24 Å	98.6º
Surface with 4 Ge (first neighbors of S)	-6.7 kcal/mol	2.27 Å	99.31º
Surface with 13 Ge (3 rings)	-9.9 kcal/mol	2.25 Å	103.1º

Table S2. Ge 3d binding energies (in eV) for pristine **2D–GeH**, **2D–GeFc$_6$**, **2D–GeFc$_{11}$** and **2D–GePh**.

**Material**	**Ge 3d**			
	Ge–S	Ge–Ge	Ge–H	Ge–O
Pristine **2D–GeH**	-	29.2	30.7	32.0
**2D–GeFc$_6$**	27.9	29.6	30.7	32.0
[a]**2D–GeFc$_{11}$**	27.6	29.8	31.0	32.0
[a]**2D–GePh**	28.0	29.3	30.8	32.2

[a]Measurements done on a FTO substrate.
